# Progressive Levels of Physical Dependence to Tobacco Coincide with Changes in the Anterior Cingulum Bundle Microstructure

**DOI:** 10.1371/journal.pone.0067837

**Published:** 2013-07-04

**Authors:** Wei Huang, Joseph R. DiFranza, David N. Kennedy, Nanyin Zhang, Douglas Ziedonis, Sanouri Ursprung, Jean A. King

**Affiliations:** 1 Center for Comparative NeuroImaging, Department of Psychiatry, University of Massachusetts Medical School, Worcester, Massachusetts, United States of America; 2 Department of Family Medicine and Community Health, University of Massachusetts Medical School, Worcester, Massachusetts, United States of America; 3 Department of Psychiatry, University of Massachusetts Medical School, Worcester, Massachusetts, United States of America; Baylor College of Medicine, United States of America

## Abstract

**Background:**

The tobacco withdrawal syndrome indicates the development of neurophysiologic dependence. Clinical evidence indicates that neurophysiologic dependence develops through a set sequence of symptom presentation that can be assessed with a new 3-item survey measure of wanting, craving, and needing tobacco, the Level of Physical Dependence (PD). This study sought to determine if advancing neurophysiologic dependence as measured by the Level of PD correlates with characteristics of white matter structure measured by Fractional Anisotropy (FA).

**Methods:**

Diffusion-MRI based FA and diffusion tensor imaging probabilistic tractography were used to evaluate 11 smokers and 10 nonsmokers. FA was also examined in relation to two additional measures of dependence severity, the Hooked on Nicotine Checklist (HONC), and the Fagerström Test for Nicotine Dependence (FTND).

**Results:**

Among smokers, FA in the left anterior cingulate bundle (ACb) correlated negatively with the Level of PD (*r* = −0.68, p = 0.02) and HONC scores (*r* = −0.65, p = 0.03), but the correlation for the FTND did not reach statistical significance (*r* = −49, p = 0.12). With advancing Levels of PD, the density of streamlines between the ACb and precuneus increased (*r* = −0.67, p<0.05) and those between the ACb and white matter projecting to the superior-frontal cortex (*r* = −0.86, p = 0.0006) decreased significantly.

**Conclusions:**

The correlations between neural structure and both the clinical Level of PD survey measure and the HONC suggest that the Level of PD and the HONC may reflect the microstructural integrity of white matter, as influenced by tobacco abuse. Given that the Level of PD is measuring a sequence of symptoms of neurophysiologic dependence that develops over time, the correlation between the Level of PD and neural structure suggests that these features might represent neuroplastic changes that develop over time to support the development of neurophysiologic dependence.

## Introduction

Imaging studies have found differences in brain structure between smokers and nonsmokers [1], [2], [3], [4], [5], [6]. Fractional anisotropy (FA) is a measure of the microstructural order in white matter. High values of FA indicate uniformly ordered structure in the imaged voxel; as more heterogeneity of structural orientation occurs, the FA decreases. Previous studies have used diffusion tensor imaging (DTI) to examine FA as related to smoking. Smokers tend to have higher FA than nonsmokers [5], [6], [7], [8]. The generally higher FA seen in smokers may reflect an increase in FA that follows the initiation of smoking, but prolonged smoking appears to reverse this effect with a progressive decline in FA with increasing pack-years of smoking [6], [7]. FA has correlated with Fagerström Test for Nicotine Dependence (FTND) scores in some studies [6], [9], but not others [5], [10], [11]. Hudkins et al [6]. reported that FA in the anterior cingulum bundle (ACb) correlated inversely with FTND scores (r = −0.64). Zhang et al. reported a negative correlation (r = −0.52) between FA and FTND score in prefrontal white matter, but only in highly dependent smokers [9]. In a small study, Paul et al. reported a non-significant negative correlation (*r* = −0.58) between FA and FTND in the corpus callosum [5]. In each of these cross-sectional studies, there was the possibility that differences between smokers and nonsmokers, or between smokers with different FTND scores, might reflect conditions that predated the onset of smoking. A stronger argument for a causal connection between structure and dependence could be made if changes in structure were shown to parallel the development of dependence.

Neurophysiologic dependence is an important feature of tobacco addiction. In smokers who do not have neurophysiologic dependence, abstinence does not trigger a desire to smoke. However, in smokers who have neurophysiologic dependence, abstinence always triggers an urge to smoke. The quality and intensity of the strongest abstinence-induced urges to smoke differs between smokers and is reflected in the terms wanting, craving, and needing [12], [13], [14]. Clinical studies indicate that neurophysiologic dependence always develops through the same sequence: level 0– no abstinence-induced urges to smoke, level 1- wanting, level 2- craving, and level 3- needing (Table 1) [12], [14], [15], [16]. The observation that neurophysiologic dependence progresses through an identical sequence of 4 levels in all smokers has important theoretical implications because it implies that the alterations in the brain that underlie neurophysiologic dependence also develop in sequence in all smokers. If this is true, it might be possible to correlate the clinical symptoms of advancing neurophysiologic dependence with changes in brain structure to identify structures that are involved with neurophysiologic dependence.

**Table 1:** The Level of Physical Dependence measure.

The Level of Physical Dependence Measure		This item describes me…			
Wanting	If I go too long without smoking, the first thing I will notice is a mild desire to smoke that I can ignore.	Not at all	A little	Pretty well	A lot
Craving	If I go too long without smoking, the desire for a cigarette becomes so strong that it is hard to ignore and it interrupts my thinking.	Not at all	A little	Pretty well	A lot
Needing	If I go too long without smoking, I just can't function right, and I know I will have to smoke just to feel normal again.	Not at all	A little	Pretty well	A lot

To enable this approach, a 3-item self-report survey measure, the Levels of Physical Dependence (PD), was developed (Table 1). The Levels of PD assesses how far neurophysiologic dependence has progressed by assessing the qualitative severity of withdrawal symptoms experienced by the tobacco user. The subject is assigned to the level that coincides with the most advanced symptom that the subject experiences during withdrawal (none, wanting, craving, or needing.) This measure identifies a smoker's stage of progression through the 4 levels of neurophysiologic dependence in >99% of cases, and has been validated against a wide range of behavioral indicators [13]. As the symptoms that are assessed by the Levels of PD measure are subjective (wanting, craving, needing), to validate it as a measure of a physical process as is implied by the name Levels of Physical Dependence, it would be helpful to compare it to a physical measure such as neural structure. Because the anterior cingulate cortex has been associated with responsivity to smoking cues and craving for nicotine [17], [18], [19], [20], [21], [22], [23], and because FA has correlated negatively with the FTND in some prior studies [6], [9]. we hypothesized that the Level of PD would correlate negatively with FA in the ACb.

The Level of PD measure is based entirely on smokers' subjective reports of tobacco withdrawal symptoms which they may experience as being either mental or physical [14]. While the FTND does not ask about wanting, craving or needing, it inquires about smokers' behaviors (number of cigarettes smoked, smoking patterns, smoking when sick in bed, etc.) that would reflect physical dependence [24], [25]. The Hooked on Nicotine Checklist (HONC) is a measure of nicotine dependence that is more discriminating than the FTND at lower levels of dependence [26], [27]. The Level of PD (3 items), the FTND (7 items) and the HONC (10 items) are conceptually distinct approaches to measuring dependence. We used all three measures in this study to assess concordance of findings.

## Methods

### Subjects

Eleven healthy smokers (9 males and 2 females) and 10 nonsmokers (6 males and 4 females) of both genders were recruited from the community via word of mouth and advertisements placed on the internet (one additional smoker was excluded from the data analysis due to our discovery of a glioma). Interested respondents were screened for eligibility by phone and then evaluated in person. Exclusionary criteria included a history of brain trauma, neurological conditions, substance abuse disorder other than tobacco abuse, mental illness requiring medication, use of psychotropic medications and any safety contraindication to scanning. Subjects had to be between 18 and 39 years of age. Nonsmokers must not have smoked more than 2 cigarettes and none in the prior year. Smokers had to smoke daily for the past year and have a lifetime history of smoking >100 cigarettes. There was no minimum daily cigarette consumption required. This prospective imaging study with human subjects was approved by the Institutional Review Boards of the University of Massachusetts Medical School. Written informed consent was obtained for all participants.

At the intake assessment subjects completed a survey that collected demographic information and smoking history, and included the Level of PD [12], [13], [16], the FTND and the HONC [28], [29].

### MR image acquisition and processing

Imaging data were acquired on a Philips Achieva 3T MR scanner. Anatomical images were acquired using a high-resolution 3-dimensional T1-weighted sequence (MPRAGE) to facilitate transformation and visualization, with a matrix size of 256×256×220, voxel size 0.98×0.98×0.6 mm. Diffusion-weighted whole brain diffusion tensor imaging (DTI) volumes were acquired using echo planner imaging (EPI) from 32 directions, with 60 axial slices of 2 mm thickness and an inplane resolution of 1.75 mm; b values of 0 and 800 s/mm^2^; repetition time TR  = 8313 ms, echo time TE  = 59 ms. Number of averages was 2 to improve the signal-to-noise ratio. Total DTI acquisition time was 9.26 minutes.

As prior studies have implicated the anterior cingulate cortex in craving [17], [18], [19], [20], [21], [22], [23], we directed our DTI analysis to the anterior cingulum bundle (ACb). Since the MNI152 space is used as the common target space for the diffusion analysis, described below, we established a right and left ACb reference region of interest (ROI) in this space. These ROI's were generated in the MNI space by manual segmentation of just the white matter contained within the confines of the cingulate gyrus, anterior to the anterior commissure.

DTI data were analyzed using the FSL collection of software tools from the Oxford Centre for Functional Magnetic Resonance Imaging of the Brain (FMRIB; www.fmrib.ox.ac.uk/fsl) [30], [31], [32], [33], Voxel-wise generation of the FA data was carried out using FSL's FDT, including eddy current correction, and generation of all tensor metrics (for FA and an eigen decomposition of the voxel-wise diffusion tensor).

The Tract-Based Spatial Statistic (TBSS) procedure [34], was performed in order to identify group FA difference. For alignment of the data into the standard MNI152 space, we used the study specific (-n) analysis option to identify the 'most representative' single subject. All subjects are non-linearly registered to this representative subject, and the representative subject is affine-aligned into MNI152 standard space. Thus every subject's image is transformed into 1x1x1mm MNI152 space by combining the nonlinear transform to the target FA image with the affine transform from that target to MNI152 space. The right and left ACb ROI's can be represented in the original subject's space through the application of the inverse combined transformation.

FA within the ACb for smokers and nonsmokers were further examined using ROI analysis. Among smokers, Spearman's correlation coefficients were evaluated between FA within the ACb and the Level of PD, the FTND and the HONC, respectively.

Probabilistic tractography (using FSL's, ‘bedpostx’ for local modeling of diffusion parameters, and ‘probtrackx’) was performed in each subject's native space seeded from the left and right ACb, respectively. This produces a map of the relative density of the probability distribution of principle diffusion direction streamlines from all voxels of the ACb-seed region to all other voxels. While not measuring “connectivity” *per se*, this measure provides a spatial distribution of the diffusion-based evidence for long-range diffusion orientation coherence. Using SPM8, these density maps were compared between smokers and nonsmokers. In addition, the density of streamline probabilities to the ACb was further correlated against the level of PD, the FTND and the HONC within the smokers.

## Results

Table 2 lists demographics and measures of nicotine dependence for smokers and nonsmokers. There was no significant age difference between smokers and nonsmokers.

**Table 2:** Demographics and measures of nicotine dependence for smokers and nonsmokers.

Subjects	Smokers (n = 11)		Nonsmokers (n = 10)	
	Mean	SD	Mean	SD
Age (years)	23.7	1.98	22.5	6.78
FTND	4.0	1.69	–	–
HONC	6.0	2.88	–	–
Level of PD	1.6	0.92	0	0

**Figure 1:**
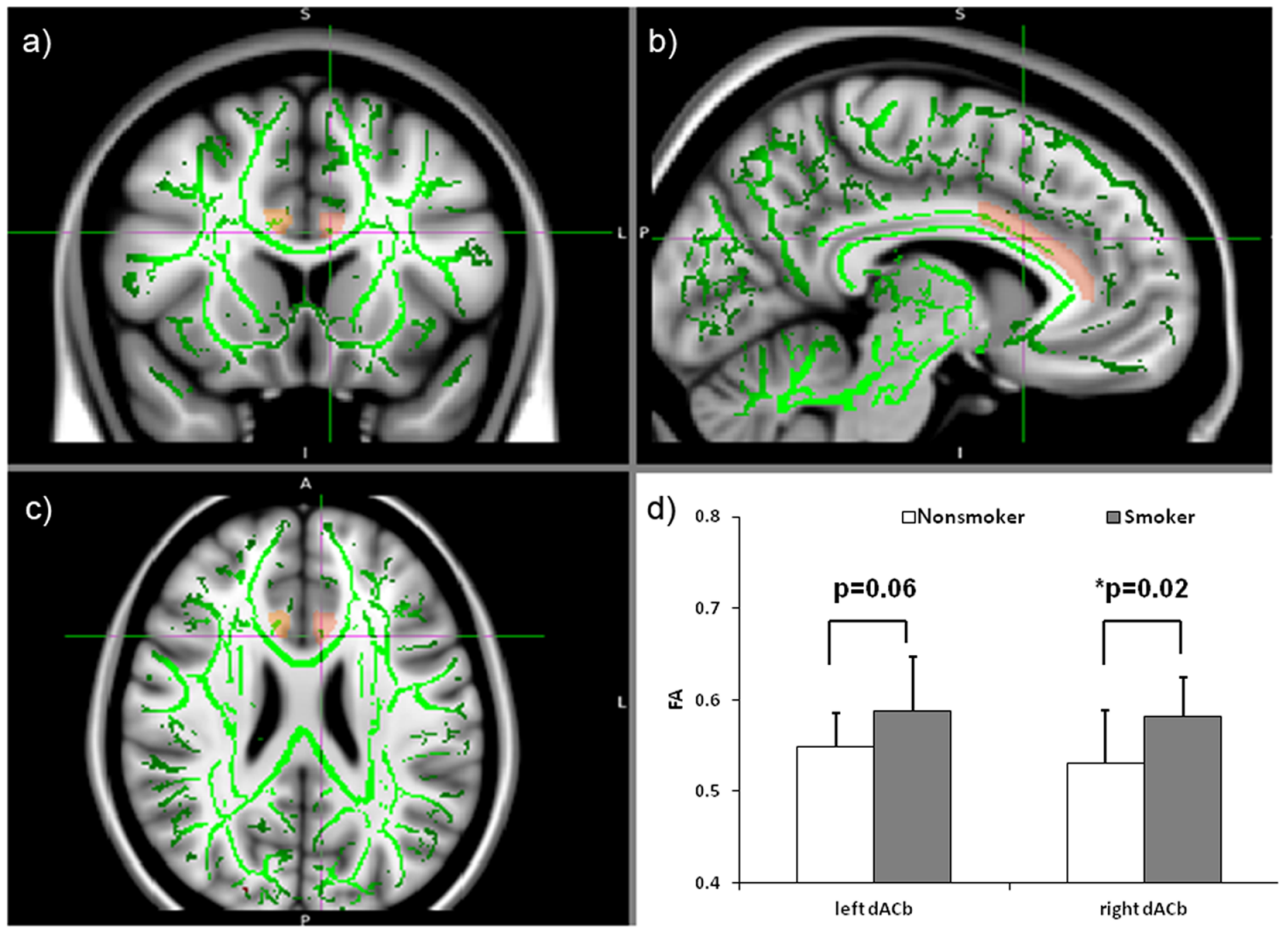
A comparison of FA in smokers and nonsmokers. The TBSS white matter skeleton (green) is overlaid on the grayscale standard brain. Shadowed red and pink areas are regions of interest, namely left ACb and right ACb. The right side of the brain is shown on the reader's left in the sagittal and coronal views. The faint crosshairs on each image a) coronal, b) sagittal, c) axial) mark the level of the sections on the other views (MNI coordinates [−6.3, 23.5, 21.9]). As depicted in the bar graph, compared to nonsmokers (n = 10), smokers (n = 11) showed significantly higher FA in the right ACb (t(19) = 2.34, p = 0.03) and a trend toward higher FA in the left ACb (t(19) = 2.0, p = 0.06).

**Figure 2:**
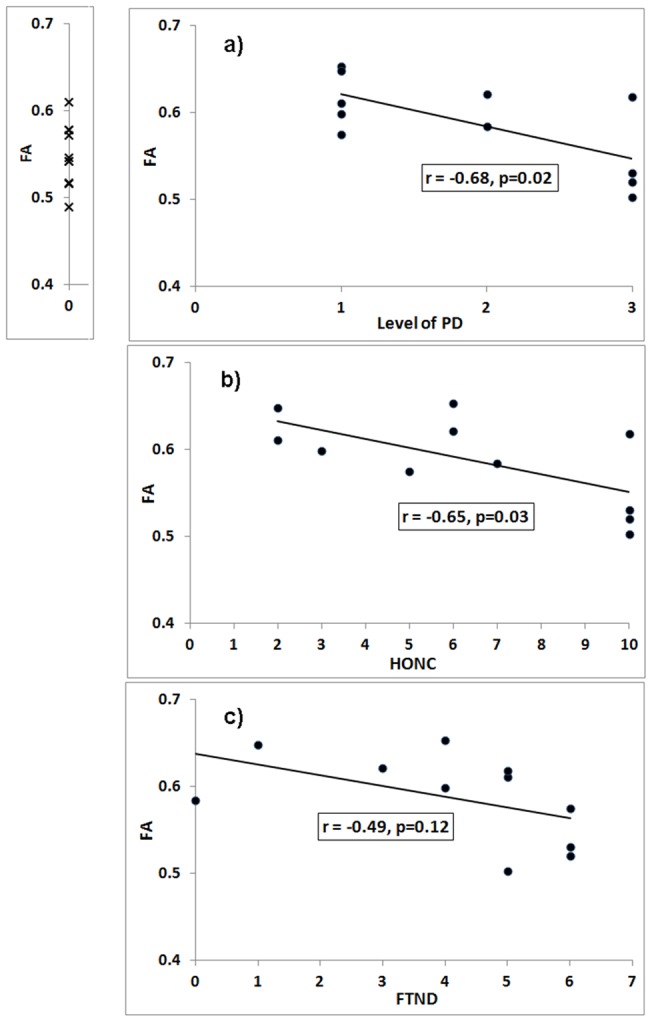
Correlation between FA and three measures of tobacco dependence. The x's on the vertical axis in the first plot indicate the FA values of each of 10 nonsmokers. The black dots in the scatter plots indicate the values for 11 individual smokers. The lines indicate the linear correlation between each measure of addiction and FA in the left ACb. a) Level of Physical Dependence (*r* = −.68, p = 0.02); b) HONC (r = −0.65, p = 0.03); c) FTND (r = −0.49, p = 0.12).

Compared to nonsmokers, smokers demonstrated higher FA in the right ACb (t(19) = 2.34, p = 0.02), and a strong trend toward higher FA within the left ACb (t(19) = 2.0, p = 0.06, Figure 1). Among smokers, the level of PD correlated negatively with FA in the left ACb (*r* = −0.68, p = 0.02, Figure 2a). HONC scores also correlated negatively with FA in the left ACb (*r* = −0.65, p = 0.03, Figure 2b). FTND scores did not correlate significantly with FA in the left ACb (*r* = −0.49, p = 0.12, Figure 2c). No significant correlations between FA and any of the nicotine dependence measures were found in the right ACb.

mokers demonstrated less density of streamlines than nonsmokers between the ACb and the orbital frontal area, the middle cingulum and posterior cingulum bundles (p<0.05, table 2D). In the left hemisphere, there was a negative correlation between the density of streamlines leading from the ACb to the white matter leading to the superior frontal and medial superior frontal areas and the Level of PD (p<0.03), the HONC (p<0.05) and the FTND (p<0.05). In the left hemisphere, there was a positive correlation between the density of streamlines between the ACb and the white matter leading to the precuneus and the Level of PD (p<0.03), the HONC (p<0.05) and the FTND (p<0.05).

**Figure 3:**
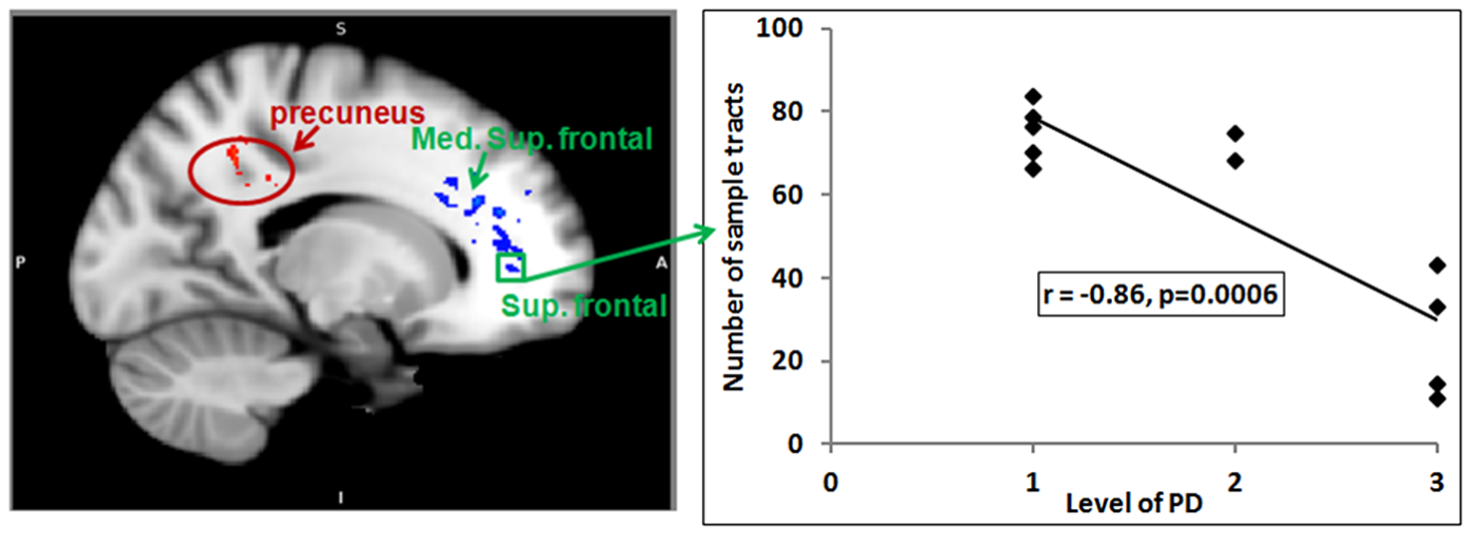
Correlation map of density of streamlines with the Level of PD in smokers. Using the ACb as a seed, the density of streamlines to other brain regions were measured and correlated with the Level of PD. Red indicates areas of positive correlation and blue indicates areas of negative correlation between streamline density and the Level of PD. In the left hemisphere, white matter streamlines approaching the superior frontal and medial superior frontal areas showed negative correlation with PD (p<0.03), whereas white matter approaching the precuneus showed positive correlation (r = .75, p<0.05). Fewer correlations were seen in the right hemisphere. The graph shows the correlation between the Level of PD and the density of streamlines between the ACb and the white matter approaching the superior frontal area indicated by the green square. Level of PD showed a strong negative correlation with streamline density (*r* = −.86, p = 0.0006).

There were many areas with statistically significant correlations for each measure of dependence, with the strength of the correlations varying from location to location. Table 3 provides coordinates and Figure 3 shows a representative brain map and correlation plot for areas of correlation with Level of PD. Fewer correlations were seen in the right hemisphere.

**Table 3:** Summary of results.

Results		Coordinates x y z	
A. Smokers showed significantly higher FA than nonsmokers in the right ACb.			p = 0.03
B. Smokers showed a strong trend toward higher FA than nonsmokers in the left ACb.			p∼0.06
C. Among smokers, FA in the left ACb correlated inversely with the…	Level of PD		*r* = –0.68, p = 0.02
	HONC		*r* = −0.65, p = 0.03
	FTND (not significant)		*r* = −0.49, p = 0.12
D. Smokers demonstrated less density of streamlines than nonsmokers between the ACb and the…	Orbital frontal area Mid cingulum bundle Posterior cingulum bundle		p<0.05
E. The Level of PD correlated negatively with the density of streamlines between the ACb and the white matter approaching the…	Superior frontal area	−13 52 −11	r = −0.86, p<0.001
		−1 53 0	
	Superior medial frontal area	4 55 7	
		−5 50 16	
		−2 65 25	
		−16 32 26	
		8 58 24	
		−16 40 22	
	Medial frontal area	3 69 8	
		3 35 35	
F. The Level of PD correlated positively with the density of streamlines between the ACb and the white matter approaching the…	Precuneus	−18 −47 38	r = 0.75, p<0.05
		0 −43 50	
		−10 −57 36	

## Discussion

Our data demonstrate a correlation between the Level of PD measure and FA in the left ACb, and correlations between the Level of PD and the density of streamlines connecting the ACb to the precuneus and superior frontal areas. While the concurrent validity of the Level of PD measure has been established against many behavioral indicators [13], [16], this is the first study to demonstrate that the Level of PD correlates with measures of brain microstructure. Our study demonstrates the utility of the Level of PD scale as a measure of the severity of neurophysiologic tobacco dependence for neuroimaging research. As a 3-item self-assessment measure, the Level of PD scale could be easily incorporated into ongoing or future studies.

While the HONC and FTND do not measure a sequence of symptoms, advancing Levels of PD are associated with increasing scores on both the HONC and FTND [16]. As scores on the HONC and FTND increased, the density of streamlines between the ACb and the precuneus increased, while the density of streamlines to the superior frontal and medial superior frontal areas decreased, replicating the results seen with the Level of PD measure. The concordance of results for both FA and the streamline probability pattern make it unlikely that the associations are artifacts produced by multiple comparisons.

A simple association between a measure of addiction and neural structure might represent a pre-existing condition that renders certain individuals more likely to become addicted. Indeed, Hong et al. have described a genetic variant of the α5 nicotinic receptor subunit that is associated with a dorsal anterior cingulate-ventral striatum/extended amygdala circuit such that the risk allele decreases the intrinsic resting functional connectivity strength in this circuit [35]. Although this trait predicted the severity of addiction in smokers, it was not secondary to smoking and was observed in both smokers and nonsmokers [35]. Consideration must be given to the possibility that FA in the ACb is a static pre-existing condition that predestines how addicted a smoker will become. The proposition that FA is a static pre-existing condition is incompatible with the observation that FA *increases* in relation to pack-years of cigarette exposure in adolescents, and then *decreases* with increasing pack-years of smoking in adults [6], [7]. Also arguing against FA as a pre-existing condition, is the strong correlation between FA in the ACb and the Level of PD. The Level of PD measure is unique among all measures of nicotine addiction in that it assesses symptoms that develop in a set sequence over time [14]. Although this was a cross-sectional study, given the sequential nature of the Level of PD measure, the most parsimonious interpretation of our data is that decreasing FA in the ACb reflects progressive neuroplasticity that supports the development of neurophysiologic dependence.

Clinical studies indicate that neurophysiologic dependence progresses in a fixed sequence of developmental stages in all tobacco users [16]. As the symptoms of tobacco withdrawal that are assessed by the Level of PD measure are subjective, some have questioned the appropriateness of labeling this “physical dependence.” In the current study, the Level of PD measure showed strong correlations (up to r = −.86) with alterations in neural structure as measured by FA and streamline density. Although the neural process that accounts for withdrawal remains unknown, these data support the proposition that the Level of PD correlates with neural features of the brain.

It is also interesting that our analyses identified the ACb as the area of maximal correlation between FA and both the Level of PD and the HONC. A number of studies indicate that the anterior cingulate cortex (ACC) is involved with craving and the response to drug cues. Activation of the ACC has been demonstrated in conjunction with opiate craving [36], and exposure to cues for smoking [17], [18], [19], [37], and cocaine [38], [39], [40], [41], [42]. Brody et al. found increased activation in the ACC when smokers were instructed to resist craving [20]. Consistent with the proposal that nicotine suppresses activity in areas involved in craving [43], Brody et al. did not see activation in the ACC when smoking cues were presented soon after subjects had smoked [20]. Likewise, Lim et al. found activation in the ACC when smoking cues were presented during abstinence, but not after smoking [19]. Brody et al. demonstrated attenuation of cue-induced craving and ACC activation in bupropion-treated smokers [44]. London et al. demonstrated that while the ACC deactivated during the performance of a working memory task in nonsmokers, it did not do so in smokers [45].

The proposition that changes in FA in the ACb represent neuroplastic changes that support addiction is supported by the observation that the density of streamlines between the ACb and other areas correlated with the Level of PD. As the Level of PD increased, the density of streamlines between the ACb and the precuneus increased, while the density of streamlines to the superior frontal and medial superior frontal areas decreased. Hartwell et al. found that the left ACC, medial prefrontal cortex and orbital frontal cortex were activated when smokers were told to resist craving while viewing smoking cues [37]. The frontal cortex is associated with top-down control and decreasing density of streamlines between the ACb and these areas are potentially linked to decreased ability to resist craving [46]. It is interesting to note that decreased FA and/or decreased gray matter volume in the frontal cortex have been observed with alcohol, cocaine, methamphetamine, tobacco and opiate dependence/abuse (see [11], for a review).

Our data indicate that as the level of PD progresses, FA in the ACb progressively decreases while its connectivity with the precuneus increases. While the precuneus has not been typically highlighted as an area related to addiction, a recent meta-analysis of fMRI studies of smoking cue reactivity found a reliable cue-reactivity effect in the precuneus [37], [47]. Taken together, our data suggest that the progression of PD may be associated with a remodeling of the ACb, which results in stronger density of streamlines with areas associated with cue-induced craving, and weaker density of streamlines with frontal areas that might be involved in resisting craving. Since the tractography demonstrates an alteration of neural tracts in smokers with different Levels of PD, it would be also interesting to investigate how functional connectivity changes with Levels of PD.

The limited studies of white matter structure in smokers have not produced consistent findings. We found that smokers had higher FA in the ACb. This finding is concordant with other studies that found higher FA in smokers in other white matter structures [5], [6], [7], [8], but conflicts with one study that found no areas of increased FA in heavy smokers but did find lower FA in the left anterior corpus callosum [11]. One theory of nicotine addiction describes several neuroplastic processes that account for different aspects of nicotine addiction such as withdrawal, craving and tolerance [43]. The disparity in findings between studies might be attributable to differences between study samples in age, race, duration of tobacco use, severity of addiction, and comorbidity. While we found distinct correlations between structure and addiction in young smokers, some differences in brain structure between older smokers and nonsmokers have not been shown to correlate with measures of addiction and may represent nonspecific toxicity related to long term smoking [8], [11].

An important contribution of this study is the demonstration that the Level of PD and HONC both correlate well with white matter microstructure. The observed correlations provide support for the validity of both scales as new tools for neuroscience researchers to use to assess tobacco addiction in imaging studies.

A limitation of this study is the small sample size. A larger sample size might have provided more heterogeneity and statistical power for other analyses. Our study differed from several prior studies in that the sample was younger, had a shorter duration of tobacco use, did not smoke heavily and showed greater heterogeneity in levels of addiction. Another limitation was that the study groups were not balanced for gender (9 male and 2 female smokers; 6 male and 4 female nonsmokers) [11]. Given the study limitations and the heterogeneity of the literature, it would be important to replicate the results of this study.

A strength of this study was the use of 3 separate validated measures of nicotine dependence which allowed us to demonstrate concordance of results. This is the first study to show correlations between the Levels of PD and the HONC and brain structure. We believe it is also the first study to demonstrate an association between the density of streamlines, as represented by probabilistic tractography of the cingulum bundle and measures of tobacco addiction. While changes in probabilistic tractography may relate to altered connectivity, care should be taken in that interpretation insofar as multiple other confounding factors can also alter the diffusion tensor pattern. Spatial resolution and its related partial volume effects affect the degree to which each voxel represents structure related to specific pathways. Since numerous underlying pathways can contribute to many voxels, altered minor crossing pathways can cause an apparent change that may be ascribed to changes in the dominant pathway. Nevertheless, the observed changes in streamline density pattern are indicative of underlying white matter alterations, and additional studies will be needed to tease apart the different causative factors.

In the hope of identifying the effects of smoking, imaging researchers commonly recruit only the most addicted smokers. A potential downside of this approach is that there may be limited variability in neural characteristics between subjects who differ very little in the severity of addiction. Based on the observations that symptoms of neurophysiologic dependence can appear quickly after the initiation of tobacco use [48], we recruited smokers covering the range from minimal to substantial dependence. This underused approach [6], produced a wide range of values for each dependence measure, optimizing the chance of identifying associations. While the Levels of PD and HONC measures were designed to be sensitive to low levels of addiction, the FTND has poorer discrimination in this population [27]. This factor might explain why the FTND showed a weaker correlation with FA in the ACb (*r* = −0.49), and the small sample size might explain why a correlation of this strength did not reach statistical significance. We recommend that future imaging studies include smokers who represent the full spectrum of dependence severity and that investigators employ dependence measures such as the Levels of PD and HONC that are well-suited for this population.

In conclusion, our study demonstrates that a simple, 3-item, self-report assessment of the Levels of PD correlates strongly with neural characteristics in brain areas that are associated with addiction. Longitudinal studies will be required to establish whether these neural characteristics represent neuroplastic changes that underlie addiction.
